# Fishery resource management with migratory prey harvesting in two zones- delay and stochastic approach

**DOI:** 10.1038/s41598-023-34130-x

**Published:** 2023-05-04

**Authors:** H. Niranjan, M. N. Srinivas, A. V. S. N. Murty, K. K. Viswanathan

**Affiliations:** 1 Department of Mathematics, School of Advanced Sciences, Vellore Institute of Technology, Vellore, Tamil Nadu 632014 India; 2 Department of Mathematical Modeling, Faculty of Mathematics, Samarkand State University, 15, University Blvd., 140104 Samarkand, Uzbekistan; 3 Department of Applied Mathematics and Informatics, Termez State University, 190100 Termez City, Uzbekistan

**Keywords:** Biological techniques, Computational biology and bioinformatics, Mathematics and computing

## Abstract

In this work, we looked at a two-zone aquatic habitat where both prey and predators can access the zones. The prey alternates between two zones at random. The growth of prey in the absence of a predator is believed to be logistic in each zone. The inner steady state is determined. Around the interior steady state, the deterministic model’s local and global stability is investigated. Furthermore, a stochastic stability study is performed in the neighbourhood of a positive steady state, using analytical estimates of population mean square fluctuations to investigate the system’s dynamics in the presence of Gaussian white noise.

## Introduction

In recent years, bio mathematics has been applied to a variety of problems in ecology and epidemiology^1,2^. Policymakers and scientists working in the fields of marine fisheries and other fields debated the economic and social benefits of marine protected zones^3^. Because it is expected that adult or juvenile migration will replace depleted fishing grounds beyond the boundaries of the marine reserve, it may be used as a defensive strategy. Extensive and uncontrolled fishing of marine fish can result in the extinction of various fish species. The development of marine reserves where fishing and other extractive activities are restricted could be one answer to these challenges. Marine reserves not only conserve species within the reserve, but they can help boost fish populations in surrounding areas. Managers must address the interaction between fish and their associated habitats in order for marine reserves to be effective.

Marine reserves are a promising alternative to traditional fisheries management methods such as catch limitations, gear restrictions, and/or effort restrictions. It is supposed to benefit fisheries by conserving spawning populations, providing a safe haven for pre-recruiting fish, and exporting biomass to nearby fishing regions. Non-fisheries benefit of marine reserves include biodiversity and ecological structure protection, biological reference regions, and non-consumption recreational opportunities. The advantages of establishing marine reserves can extend beyond the protection of a single overfished fish stock. Marine reserves can protect the marine environment from damage caused by destructive fishing methods, as well as gives an essential opportunity to learn about marine ecosystems. It aids in the study of species dynamics and the development of management strategies for the conservation of all aspects of a marine community. As a tool for conservation and marine environmental management, marine reserves have yielded numerous benefits^4,5^ and has done substantial research on the best management of renewable resources as a fishery, which has a direct link to sustainable development. A dynamic model for fishery resources and showed that the use of diesel-powered trawling may lead to the extinction of predator and prey species if trawling efficiency in the catch of prey species is improved, discussed the economic and biological management of renewable resources^6–11^. It is looked at the issue of two rival fish species being harvested at the same time^12^. A model for studying the taxation-based regulation of single-species fisheries was proposed^13^. A method for determining the best harvesting policy for two interdependent populations in a Lotka-Volterra environment was presented^14^. A resource-based competitive system in three species and came up with conditions for the system’s persistence and global stability^15^. The uniqueness of limit cycles in a harvested predator–prey system with Holling type III functional response was investigated^16^.

The exploitation of a single species and demonstrated that the time-dependent logistic equation with the periodic coefficient has a single positive periodic solution that is globally asymptotically stable for positive solutions^17^ and in a prey-predator fishery, it has been provided a mathematical model of nonselective harvesting^18^. They went on to define taxation as a control device for regulating a prey-predator fishery in their subsequent work^19^. It was also proposed an ideal for studying selective harvesting in a prey-predator fishery, which included a time delay in the harvesting period^20^. A dynamic model created for a single-species fishery that is largely reliant on a supply that is growing logistically^21^. They discovered that when the biomass density of the resource grows, so does the equilibrium density and maximum sustainable output of the fish population. Few researchers developed a harvested population model with diffusional migration recently^22,23^ and proposed a mathematical methodology for studying the impact of a reserved zone on an aqua ecosystem's dynamics^24^. The tools for simulating species movement between zones was presented and it has been found that there is an arrangement in which species interact successfully through adaptations to the point that predators become extinct in each patch if adaptations are not made^25^ Several mathematical tools to analyze the potential impact of establishing marine sheltered zones on aqua ecosystems, concluding that establishing a marine sheltered zone can result in significant species decline^26^. Many of the benefits associated with marine protected zones have been well investigated, and the field is a hotbed of theoretical ecology and mathematical biology study^27^. The work was presented on the stability of three and four species with migration, bionomic equilibrium, and the best harvesting strategy^28^.

On the basis of the density effect of fish populations, a unique model of fishing effort was created^29^. To characterise a number of widely used fisheries management strategies, they developed new differential equations. The results show that a control parameter (the amount of the effect of fish population size on fishing effort function) controls not only the rate of population equilibrium but also the equilibrium values. They developed a new fishing effort model by contrasting several strategies. In the case of proportional harvesting, increases in β (the noise amplitude) will cause the population to stabilise more quickly. In proportional threshold harvesting, an increase in β will accelerate the convergence to a stable equilibrium point above the equilibrium solution. Due to the periodic nature of proportional harvesting rate, which alters the value of stable equilibrium point, seasonal harvesting strategy has a higher stable equilibrium point than proportional harvesting strategy.

In order for future generations to benefit from fishing, its long-term viability must be ensured through fisheries management. The best harvesting strategy, however, typically maximises a harvester's economically significant objective function, which could result in the extinction of the resource population. As a result, achieving sustainability has proven to be much more challenging than initially anticipated; overfishing is causing catches to decline and fish populations to become increasingly limited. During his research, he developed an efficient harvesting method and formula to maximise the net profit from harvesting.

Constant harvesting and periodic harvesting are the two logistic growth models that have been used. When overcrowding and resource rivalry are taken into account^30^, the logistic growth model is adequate for animal population growth. They wanted to determine which fish harvesting techniques would result in the highest ongoing production. Second, the analysis forecast the ideal harvesting quantity that can guarantee a steady supply of tilapia fish. The optimal harvesting technique for the chosen fish farm, according to findings that were compared between the two strategies, is periodic harvesting. An ongoing seasonal harvesting approach can increase output and accelerate investment payback. Fish farming does not have enough time to rebuild the fish population because of the continual harvesting.

The management of fisheries involves taking into account how harvesting may affect the environment^31^. While fishermen strive to feed an expanding human population, some fish species have been dreadfully dwindling as a result. It’s crucial to strike a balance between ecological and economic needs. There were several deterministic models of fishing populations examined. With a constant harvest rate as well as time-dependent harvesting, three different logistic models—a straightforward one, a skewed one with a quadratic term, and one that illustrates the Allee effect—have all been taken into account. The optimal harvest rate for each population density scenario was identified using optimization and numerical computations.

A mathematical model was developed to study the dynamics of a fishery resource system in an aquatic environment that consists of two zones: a free fishing zone and a reserve zone where fishing is strictly prohibited^21^. Biological and bionomic equilibria of the system are obtained, and criteria for local stability, instability and global stability of the system were derived. It was shown that even if fishery was exploited continuously in the unreserved zone, fish populations can be maintained at an appropriate equilibrium level in the habitat. An optimal harvesting policy is also discussed using the Pontryagin’s Maximum Principle. Under continuous harvesting of fish species outside the reserved zone, fish population may be maintained at an appropriate equilibrium level. Optimal harvesting policy has been discussed and concluded that high interest will cause high inflation rate. Analyzed a mathematical model to study the dynamics of a fishery resource system with stage structure in an aquatic environment that consists of two zones namely unreserved zone (fishing permitted) and reserved zone (fishing is strictly prohibited)^32^. In this model they introduce a stage structure in which predators are split into two kinds as immature predators and mature predators. It is assumed that immature predators cannot catch the prey and their foods are given by their parents (mature predators). It is also assumed that the fishing of immature predators prohibited in the unreserved zone and predator species are not allowed to enter inside the reserved zone. The local and global stability analysis has been specified. Biological and bionomical equilibrium points of the system were derived. Mathematical formulation of the optimal harvesting policy was given and its solution was derived in the equilibrium case by using Pontryagin’s maximum principle. The vital role of reserved zone in aquatic environment for protection of fishery resources from its overexploitation is discussed by several researchers. The use of marine protect area on both biological and economical aspect was studied^33,34^. They investigated the impacts of the creation of Marine Protected Areas (MPAs), in both economic and biological perspectives. The economic indicator was assumed to be optimally managed. The biological indicator was taken as the stock density of the source. The basic fishery model was serving as the benchmark in comparing results with those that are derived from a model of two patchy populations. A crucial characteristic is the migration coefficient which describes biological linkages between protected and unprotected areas. Both economic and biological criteria are enhanced, after introducing a marine protected area, was presented. A prey-predator fishery model with the influence of prey reserve were developed and also noted that the fish population maintains at an equilibrium level in absence or presence of predator provided the population in the unreserved area lies in a certain interval^35^. The result obtained was a high interest rate will cause a high inflation rate. Bio-economic model of a prey-predator fishery with protected area and also discussed the system numerically and observed that marine protect area can be used as an effective management tool to improve resource rent under a number of circumstances^36,37^. Bionomic equilibrium and optimal harvesting^39^ is one of the interesting and informative studies on fishery models. Mathematical models (including stochastic and diffusive models) are powerful tool to interpret and catch up the dynamics exhibited by fishery model in multi zone environment. One of such model, which is studied by Srinivas et.al^39^ is the basic motive for the current work, which we analyzed the dynamics of stochastic system both analytically and graphically and parametric variations with the help of graphical solutions. One of the interesting objectives of the work is the stochastic stability of a prey-predator system in a two-zone aqua environment with logistic growth patterns. It has been prompted us to use analytical tools to investigate the stochastic stability of an aqua environment^29,38^.

## Mathematical model

The following system of four nonlinear ordinary differential equations is the stochastic model of a prey-predator fishery resource:

1 $$x^{\prime}(t) = \phi_{1} \left( {x,z} \right)x + \sigma_{2} y + \beta_{1} \xi_{1} (t)$$

2 $$y^{\prime}(t) = \sigma_{1} x + \phi_{2} \left( {y,w} \right)y + \beta_{2} \xi_{2} (t)$$

3 $$z^{\prime}(t) = \phi_{3} \left( {x,z} \right) + \beta_{3} \xi_{3} (t)$$

4 $$w^{\prime}(t) = \phi_{4} \left( {y,w} \right) + \beta_{4} \xi_{4} (t)$$

where $$\phi_{1} (x,z) = r - \sigma_{1} - q_{1} E_{1} - m_{1} z - (rx/K)$$; $$\phi_{2} (y,w) = s - \sigma_{2} - q_{2} E_{2} - m_{2} w - (sy/L)$$

$$\phi_{3} (x,z) = \alpha_{1} z - \left( {\alpha_{1} z^{2} /\gamma_{1} x} \right)$$; $$\phi_{4} (y,w) = \alpha_{2} w - \left( {\alpha_{2} w^{2} /\gamma_{2} y} \right)$$.

**Figure 1:**
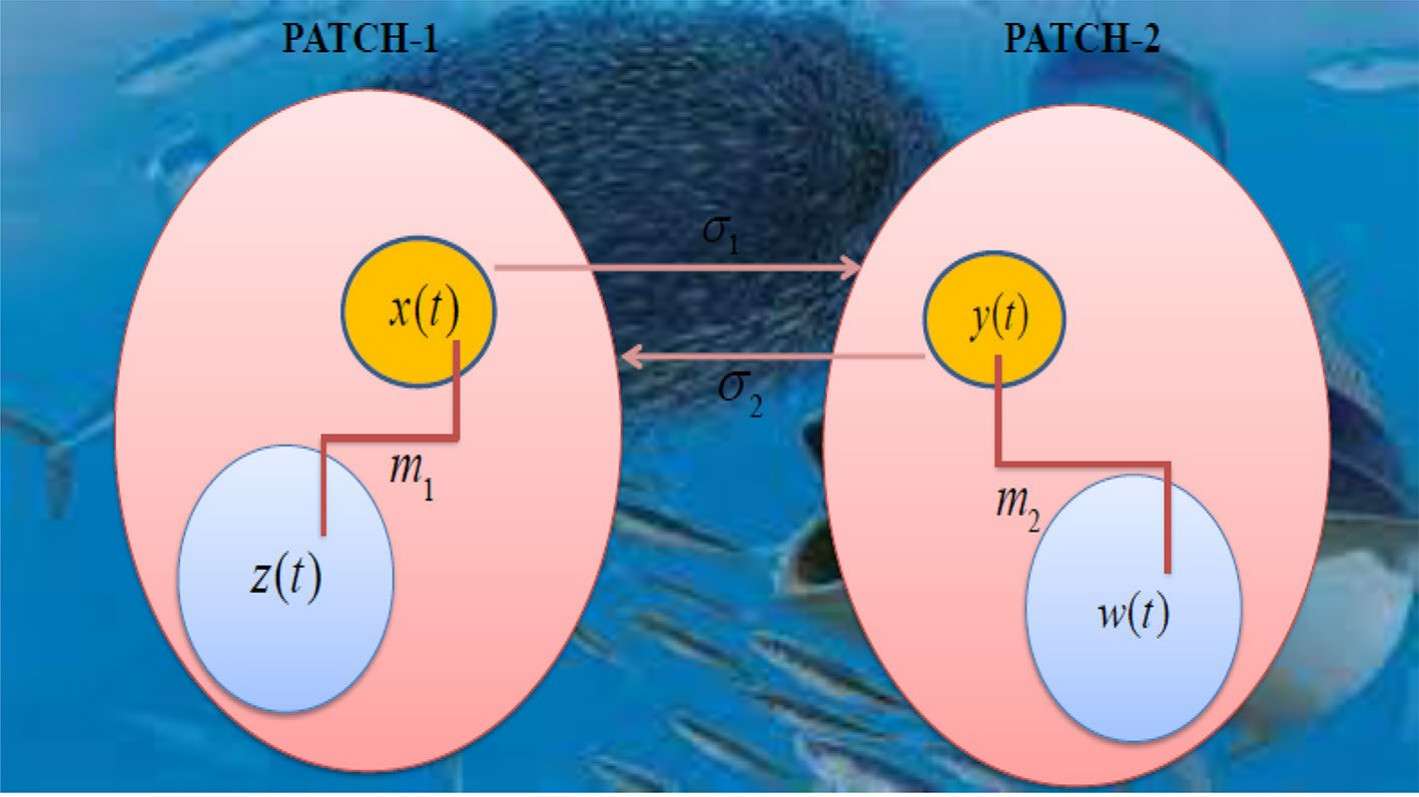
Schematic representation of the mode (l)–(4).

The following qualities are presumptions made by the model: It is a prey-predator system in a two-patch habitat. Both regions are accessible to predators and prey. Each patch must have a consistent appearance. The prey is believed to randomly move between the two regions. The growth of prey in each patch is thought to be logistic in the absence of predators. With inherent growth rates $\alpha_{1}$ and $\alpha_{2}$ carrying capacities related to population size, the predator consumes the food in the area and grows exponentially as the population grows. In patch-1, $x(t)$, $z(t)$, $E_{1}$, $K$, $\gamma_{1}$, $m_{1}$, $q_{1}$, $r$, $\alpha_{1}$ represents the biomass density of prey species, predator species, effort applied to harvest the fish population, carrying capacity of prey species, equilibrium ratio of prey to predator biomass, prey mortality rate due to predation, catch ability coefficient, intrinsic growth rate of prey species, and intrinsic growth rates of predators, in that order. In patch-2, the symbols $y(t)$, $w{(}t{)}$, $E_{2}$, $L$, $\gamma_{2}$, $m_{2}$, $q_{2}$, $s$, $\alpha_{2}$ for biomass density of prey species, biomass density of predator species, effort put forth to deplete the fish population, carrying capacity of prey species, equilibrium ratio of prey to predator biomass, prey mortality rate due to predation, catchability coefficient, intrinsic growth rate of prey species, intrinsic growth rate of predator species, and catchability coefficient, respectively, are represented. $\sigma_{1}$, $\sigma_{2}$ represents the migration rates from patch -1 to patch-2 and vice-versa. $\beta_{i} ,i = 1,2,3,4$ represents the amplitude of noise on the species prey1, prey2, predator1, predator2 respectively. $\xi \left( t \right)$ $=$ $\left[ {\xi_{1} (t),\xi_{2} (t),\xi_{3} (t),\xi_{4} (t)} \right]$ is a four-dimensional Gaussian white noise process satisfying $E\left[ {\xi_{i} \left( t \right)} \right] = 0;$ $E\left[ {\xi_{i} \left( t \right)\xi_{j} \left( {t^{\prime}} \right)} \right] = \delta_{ij} \delta \left( {t - t^{\prime}} \right);\,i,j = 1,2,3,4$ where $\delta_{i\,j}$ is the Kronecker delta function and $\delta$ is the Dirac delta function. The proposed model is a multi-zonal (patch-1, patch-2) ecological environment, where prey and predator populace exist in two patches named as patch-1 and patch-2. There is an interaction among the species of both the patches is migration. Migration of prey species is allowed from patch-1 to patch-2 and vice-versa. The pictorial representation of the proposed model including migration coefficients $m_{1} \,\,and\,\,m_{2}$ is presented as a schematic diagram, which is labeled as Fig. 1 is as follows. Similarly, all the attributes involved in the proposed system (1)–4) are described and framed with best fit of values given in Table 1.

**Table 1:** Physical description of the parameters in the stochastic model (1)–(4).

Parameters	Description of the parameters	Values
$\sigma_{1}$	Migration rate from patch-1 to patch-2	1.8
$\sigma_{2}$	Migration rate from patch-2 to patch-1	1.6
$\alpha_{1}$	Inherent growth rate of predator-1	0.5
$\alpha_{1}$	Inherent growth rate of predator-2	0.8
$r$	Intrinsic growth rate of prey in patch-1	3
$s$	Intrinsic growth rate of prey in patch-2	2.8
$q_{1}$	Catch ability coefficient of prey in patch-1	0.5
$q_{2}$	Catch ability coefficient of prey in patch-2	0.7
$m_{1}$	Interaction rate of prey and predator in patch-1	1.2
$m_{2}$	Interaction rate of prey and predator in patch-2	1.8
$\gamma_{1}$	Equilibrium ratio of prey to predator in patch-1	0.5
$\gamma_{2}$	Equilibrium ratio of prey to predator in patch-2	0.3
$E_{1}$	Effort applied to harvest the prey in patch-1	2.5
$E_{2}$	Effort applied to harvest the prey in patch-2	3.5
$K$	Carrying capacity of prey in patch-1	10
$L$	Carrying capacity of prey in patch-2	15
$\beta_{i}$	Intensities of each individual population	Variable
$\xi_{i}$	Gaussian white noise of each individual population	Variable

### Equilibrium analysis without noise

The interior steady state of the system in the absence of noise (i.e. $\beta_{i} = 0,i = 1,2,3,4$) is denoted by $P(x\,*,y\,*,z\,*,w\,*)$, where $x\,*, \, y\,*, \, z\,*, \, w*$ are positive solutions of the following equations:

5 $$x^{\prime}(t) = y^{\prime}(t) = z^{\prime}(t) = w^{\prime}(t) = 0$$

After eliminating $z*$ and $w*$,

6 $$y\,* = \left[ {x*(E_{1} q_{1} + \sigma_{1} - r) + (x\,*)^{2} \left( {\gamma_{1} m_{1} + (r/K)} \right)} \right]/\sigma_{2}$$

Eliminating $y\,*$ from (5) and (6), we get

7 $$a_{3} (x\,*)^{3} + b_{3} (x\,*)^{2} + c_{3} x* + d_{3} = 0$$

where $$a_{3} = \left[ {\left( {m_{1} \gamma_{1} + (r/K)} \right)^{2} \left( {(s/L) + m_{2} \gamma_{2} } \right)} \right]/\sigma_{2}^{2}$$; $$b_{3} = - 2\left[ {\left( {r - \sigma_{1} - q_{1} E_{1} } \right)\left( {(s/L) + m_{2} \gamma_{2} } \right)\left( {m_{1} \gamma_{1} + (r/K)} \right)} \right]/\sigma_{2}^{2}$$; $$c_{3} = \, \frac{1}{{\sigma_{2}^{2} }}\left( {\left( {r - \sigma_{1} - q_{1} E_{1} } \right)^{2} (s/L) + m_{2} \gamma_{2} } \right)$$$$- \, \left( {m_{1} \gamma_{1} + (r/K)} \right)\left( {s - \sigma_{2} - q_{2} E_{2} } \right)/\sigma_{2}$$ $$d_{3} = \left\{ {\left( {s - \sigma_{2} - q_{2} E_{2} } \right)\left( {r - \sigma_{1} - q_{1} E_{1} } \right)/\sigma_{2} } \right\} - \sigma_{1}$$

Equation (7) has a unique positive solution if the following inequalities hold.

7a $$\left( {r - \sigma_{1} - q_{1} E_{1} } \right)^{2} \left( {sL^{ - 1} + m_{2} \gamma_{2} } \right)\, < \left( {m_{1} \gamma_{1} + rK^{ - 1} } \right)\sigma_{2} \left( {s - \sigma_{2} - q_{2} E_{2} } \right)$$

7b $${\text{and}}\,\left( {s - \sigma_{2} - q_{2} E_{2} } \right)\left( {r - \sigma_{1} - q_{1} E_{1} } \right){ < }\sigma_{1} \sigma_{2}$$

Also, $w* = \gamma_{2} \,y*$, $z* = \gamma_{1} x*$; For $y*$ to be positive, we must have $(x*) > \left( {r - \sigma_{1} - q_{1} E_{1} } \right)\left( {m_{1} \gamma_{1} + rK^{ - 1} } \right)^{ - 1}$.

### Steadiness without noise

Let's pretend that the given system has a single positive equilibrium point $P\,(x\,*,y\,*,z\,*,w\,*)$, and we're looking at the dynamics of the system around it. In the absence of noise, the characteristic equation of the Jacobian matrix of the system (1)–(4) is given by

8 $$\lambda^{4} + a_{11} \lambda^{3} + a_{22} \lambda^{2} + a_{33} \lambda + a_{44} = 0$$

where

$$a_{11} = \alpha_{1} + \alpha_{2} + b + a > 0;a_{22} = \alpha_{1} \alpha_{2} + b\alpha_{1} + b\alpha_{2} + ab + a\alpha_{1} + m_{1} \alpha_{1} \gamma_{1} x* - \sigma_{1} \sigma_{2} + a\alpha_{2} + m_{2} \alpha_{2} \gamma_{2} y* > 0$$

$$a_{33} = a_{1} \alpha_{1} \alpha_{2} + b\alpha_{1} \alpha_{2} + ab\alpha_{1} + ab\alpha_{2} + \alpha_{1} \alpha_{2} m_{2} \gamma_{2} y* + \alpha_{1} \alpha_{2} m_{2} \gamma_{2} y* + am_{2} \alpha_{2} \gamma_{2} y* - \alpha_{1} \sigma_{1} \sigma_{2} - \alpha_{2} \sigma_{1} \sigma_{2}$$

$$m_{1} x*b\alpha_{1} \gamma_{1} + m_{1} x*\alpha_{1} \alpha_{2} \gamma_{1} > 0$$

$$\begin{aligned} a_{44} & = \frac{{\alpha_{1} \alpha_{2} r\sigma_{1} (x*)^{2} }}{K\,y*} + \frac{{\alpha_{1} \alpha_{2} s\,y*}}{L}\left( {\frac{r\,x*}{K} + \frac{{\sigma_{2} \,y*}}{x*}} \right) + \frac{{\alpha_{1} \alpha_{2} r\sigma_{1} (x*)^{2} }}{Ky*} + \left( {\frac{{\sigma_{2} \alpha_{1} \alpha_{2} m_{2} \gamma_{2} (y\,*)^{2} }}{{x^{*} }} + \frac{{r\alpha_{1} \alpha_{2} m_{2} \gamma_{2} x*y*}}{K}} \right) \\ & \quad + \left( {\frac{{\sigma_{1} \alpha_{1} \alpha_{2} m_{1} \gamma_{1} (x*)^{2} }}{{y^{*} }} + \frac{{s\alpha_{1} \alpha_{2} m_{1} \gamma_{1} x*y*}}{L}} \right) + m_{1} m_{2} \alpha_{1} \alpha_{2} \gamma_{1} \gamma_{2} x*y* > 0 \\ \end{aligned}$$

$${\rm where}, a = \, \frac{{\sigma_{2} \,y*}}{x*} + \frac{r\,x*}{K}; b = \, \frac{{\sigma_{1} x*}}{y*} + \frac{s\,y*}{L}$$

According to Routh–Hurwitz criteria, if $a_{11} > 0$, $a_{33} > 0$, $a_{44} > 0$, $a_{33} (a_{11} a_{22} - a_{33} ) > a_{11}^{2} a_{44}$ and $a_{44} (a_{11} a_{22} a_{33} - a_{11}^{2} a_{44} - a_{33}^{2} ) > 0$ then the Eq. (8) has negative real roots. Because the aforementioned conditions are met, the interior equilibrium point $P(x\,*,y\,*,z\,*,w\,*)$ is locally stable. Using the Lyapunov theorem, we will now explore the global stability of the interior equilibrium point $P(x\,*,y\,*,z\,*,w\,*)$ of the system (1)–(4) in the absence of noise.

#### Theorem 1

If $A < x < B$ and $C < y < D$ then the interior equilibrium point $P(x\,*,y\,*,z\,*,w\,*)$ is globally asymptotically stable

In which

$$A = \frac{1}{{m_{1} }}\left[ { - \sqrt {\frac{4r}{{m_{1} K\gamma_{1} }} + \frac{{4r^{2} }}{{m_{1}^{2} K^{2} \gamma_{1}^{2} }}} + 1 + \frac{2r}{{m_{1} K\gamma_{1} }}} \right] > 0$$

$$B = \frac{1}{{m_{1} }}\left[ { + \sqrt {\frac{4r}{{K\gamma_{1} m_{1} }} + \frac{{4r^{2} }}{{K^{2} \gamma_{1}^{2} m_{1}^{2} }}} + 1 + \frac{2r}{{K\gamma_{1} m_{1} }}} \right] > 0$$

$$C = \frac{{\sigma_{1} \,x*}}{{m_{2} \sigma_{2} \,y*}}\left[ { - \sqrt {\frac{4s}{{L\gamma_{2} m_{2} }} + \frac{{4s^{2} }}{{L^{2} \gamma_{2}^{2} m_{2}^{2} }}} + 1 + \frac{2s}{{L\gamma_{2} m_{2} }}} \right] > 0$$

$$D = \frac{{\sigma_{1} \,x*}}{{m_{2} \sigma_{2} \,y*}}\left[ { + \sqrt {\frac{4s}{{L\gamma_{2} m_{2} }} + \frac{{4s^{2} }}{{L^{2} \gamma_{2}^{2} m_{2}^{2} }}} + 1 + \frac{2s}{{L\gamma_{2} m_{2} }}} \right] > 0$$

#### Proof

The following positive definite function $V(x,y,z,w)$ in the neighborhood of $P(x^{*} ,y^{*} ,z^{*} ,w^{*} )$ is considered and $V(x*,y*,z*,w*) = 0$.

$$V = f_{1} (x,x^{*} ) + l_{1} f_{2} (y,y^{*} )+ l_{2} f_{3} (z,z^{*} ) + l_{3} f_{4} (w - w^{*} )$$

$${\rm where}\,f_{1} (x,x*) = x - x* - x*\ln \left( {x/x*} \right)$$; $$f_{2} (y,y^{*} ) = y - y* - y*\ln \left( {y/y*} \right)$$$$f_{3} (z,z*) = z - z* - z*\ln \left( {z/z\,*} \right)$$; $$f_{4} (w,w\,*) = w - w\,* - w\,*\ln \left( {w/w\,*} \right)$$

The time derivative of $V$ is given by $V^{\prime}(t) = \frac{(x - x*)}{x}x^{\prime}(t) + l_{1} \frac{(y - y*)}{y}y^{\prime}(t) + l_{2} \frac{(z - z*)}{z}z^{\prime}(t) + l_{3} \frac{(w - w\,*)}{w}w^{\prime}(t)$.choosing $l_{1} = \frac{y*}{{x*}}\frac{{\sigma_{2} }}{{\sigma_{1} }}$; $l_{2} = \frac{1}{{\alpha_{1} }}$; $l_{3} = \frac{1}{{\alpha_{2} }}$,

we get, $$V^{\prime}\left( t \right) = - \frac{s}{L}\frac{y*}{{x*}}\frac{{\sigma_{2} }}{{\sigma_{1} }}\left( {y - y*} \right)^{2} - \frac{r}{k}(x - x*)^{2}$$

$$- m_{1} \left( {x - x*} \right)\left( {z - z*} \right)- \frac{{\sigma_{2} }}{{\sigma_{1} }}\frac{y*}{{x*}}m_{2} \left( {w - w*} \right)(y - y*) - \frac{{\sigma_{2} }}{{\sigma_{1} }}\frac{y*}{{x*}}m_{2} (w - w\,*)(y - y\,*)$$

$$- \frac{{\sigma_{2} }}{{x^{*} yx}}\left( { - xy* + yx*} \right)^{2}- \frac{1}{{\gamma_{1} x}}(z - z\,*)^{2} + \frac{1}{x}(z - z\,*)(x - x\,*) - \frac{1}{{\gamma_{2} y}}(w - w\,*)^{2}+ \frac{1}{y}(w - w\,*)(y - y\,*)$$

To be negative $V^{\prime }$, we must have $A < x < B$ and, $C < y < D$ which are both provided in the theorem’s expression. As a result, if prey populations fall within a given range in the presence of predators and harvesting, they can be maintained at an optimum equilibrium level.

The above theorem indicates that, in the presence of predators, populations may be sustained at an appropriate equilibrium level if the populations in the both the patches lie in a certain interval.

### Stability analysis with noise

The effect of random fluctuations in the environment cannot be included in the approximations generated from deterministic models. The stochastic analysis allows us to investigate the dynamics of any natural ecosystem that is subjected to random environmental changes. The parameters of the system bounce about their mean values in the stochastic model (1–4). As a result, the previously set equilibrium point will now oscillate about the mean state. The random noise is included into the model as additive Gaussian white noise, and thus any system parameter $p$ reduces to $p + \beta \xi (t)$, where $\beta \in {\mathbb{R}}$ is the noise amplitude and is the $\xi (t)$ Gaussian white noise process. Because the goal of this study is to visualize the dynamics of the system around the interior equilibrium point, we use the perturbation approach to linearize the model.

9 $${\text{Let}}\,x(t) = u_{1} (t) + S^{*} \,;y(t) = u_{2} (t) + P^{*} ;\,z(t) = u_{3} (t) + T^{*} ;w(t) = u_{4} (t) + U^{*}$$

Then we have,

10 $$x^{\prime}(t) = u_{1}^{\prime } (t);y^{\prime}(t) = u_{2}^{\prime } (t);z^{\prime}(t) = u_{3}^{\prime } (t);w^{\prime}(t) = u_{4}^{\prime } (t)$$

Using the above perturbations, we identify the linear system of the model (1)–(4) as

11 $$\begin{aligned} u^{\prime}_{1} (t) & = - m_{1} S^{*} u_{3} (t) - N_{1} S^{*} u_{1} (t) + \beta_{1} \xi_{1} (t) \\ u^{\prime}_{2} (t) & = - m_{2} P^{*} u_{4} (t) - N_{2} P^{*} u_{2} (t) + \beta_{2} \xi_{2} (t) \\ u^{\prime}_{3} (t) & = (N_{3} T^{{*^{2} }} /S^{{*^{2} }} )u_{1} (t) + ( - N_{3} T^{*} /S^{{*^{2} }} )u_{3} (t) + \beta_{3} \xi_{3} (t) \\ u^{\prime}_{4} (t) & = (N_{4} U^{{*^{2} }} /P^{{*^{2} }} )u_{2} (t) + ( - N_{4} U^{*} /P^{{*^{2} }} )u_{4} (t) + \beta_{4} \xi_{4} (t) \\ \end{aligned}$$

Here $$N_{1} = r/K;N_{2} = s/L;N_{3} = \alpha_{1} /\gamma_{1} ;N_{4} = \alpha_{2} /\gamma_{2}$$.

Applying Fourier transform on the linear system, we get the following algebraic system

12 $$\begin{aligned} \beta_{1} \tilde{\xi }_{1} (\omega ) & = (i\omega + N_{1} S^{*} )\tilde{u}_{1} (\omega ) + m_{1} S^{*} \tilde{u}_{3} (\omega ) \\ \beta_{2} \tilde{\xi }_{2} (\omega ) & = (i\omega + N_{2} P^{*} )\tilde{u}_{2} (\omega ) + m_{2} P^{*} \tilde{u}_{4} (\omega ) \\ \beta_{3} \tilde{\xi }_{3} (\omega ) & = \left( {i\omega + (N_{3} T^{*} )/S^{{*^{2} }} } \right)\tilde{u}_{3} (\omega ) - N_{3} \tilde{u}_{1} (\omega )(T^{{*^{2} }} /S^{{*^{2} }} ) \\ \beta_{4} \tilde{\xi }_{4} (\omega ) & = \left( {i\omega + (N_{4} U^{*} /P^{{*^{2} }} )} \right)\tilde{u}_{4} (\omega ) - N_{4} (U^{{*^{2} }} /P^{{*^{2} }} )\tilde{u}_{2} (\omega ) \\ \end{aligned}$$

The above system can be represented in the matrix form as

13 $$M\left( \omega \right)\tilde{u}\left( \omega \right) = \tilde{\xi }\left( \omega \right)$$

Here, $$M\left( \omega \right) = \left( {m_{ij} (\omega )} \right)_{{4{\text{x}}4}} \,;$$ $$\tilde{u}\left( \omega \right) = \left( {\tilde{u}_{j} (\omega )} \right)_{{1{\text{x4}}}} \,\,;\,\tilde{\xi }\left( \omega \right) = \left( {\beta_{j} \tilde{\xi }_{j} (\omega )} \right)_{{1{\text{x}}4}} \,$$

$$\begin{aligned} m_{11} & = (N_{1} S^{*} + i\omega );\,m_{21} = 0;\,m_{31} = - (N_{3} T^{{*^{2} }} /S^{{*^{2} }} )m_{41} = 0; \\ m_{12} & = 0;m_{22} = (N_{2} P^{*} + i\omega );\,m_{32} = 0;m_{42} = - (N_{4} U^{{*^{2} }} /P^{{*^{2} }} ); \\ m_{13} & = m_{1} S^{*} ;;\,m_{23} = 0;\,\,m_{33} = (N_{3} T^{*} /S^{{*^{2} }} ) + i\omega ;m_{43} = 0; \\ m_{14} & = 0;m_{24} = m_{2} P^{*} ;m_{34} = 0;m_{44} = (N_{4} U^{*} /P^{{*^{2} }} ) + i\omega ; \\ \end{aligned}$$

Equation (13) can also be written as $\tilde{u}\left( \omega \right) = G(\omega )\tilde{\xi }\left( \omega \right)$.

where, $$G(\omega ) = \left[ {M\left( \omega \right)} \right]^{ - 1} = Adj\,M\left( \omega \right)/\left| {M\left( \omega \right)} \right| = \left( {g_{ij} } \right)_{{4{\text{x}}4}}$$

We now describe some of the basics of the random population function. If the function $Y(t)$ has a zero mean value, then the fluctuation intensity (variance) of its components in the frequency interval $\left[ {\omega ,\omega + d\omega } \right]$ is $S_{Y} (\omega )d\omega$, where $S_{Y} (\omega )$ is spectral density of $Y$ and is defined as

14 $$S_{Y} (\omega ) = \mathop {\lim }\limits_{{\tilde{T} \to \infty }} \left[ {\left( {\left| {\tilde{Y}\left( \omega \right)} \right|^{2} } \right)/\tilde{T}} \right].$$

If $Y$ has a zero-mean value, the inverse transforms of $S_{Y} (\omega )$ is the auto covariance function

15 $$C_{Y} (\tau ) = \left\{ {\int\limits_{ - \infty }^{\infty } {S_{Y} \left( \omega \right)} e^{i\omega \tau } d\omega } \right\}/2\pi$$

The corresponding variance of fluctuations in $Y(t)$ is given by

16 $$\sigma_{Y}^{2} = C_{Y} (0) = \left\{ {\int\limits_{ - \infty }^{\infty } {S_{Y} (\omega )} d\omega } \right\}/2\pi$$

and the auto correlation function is the normalized auto covariance as

17 $$P_{Y} (\tau ) = [C_{Y} (\tau )]/[C_{Y} (0)]$$

For a Gaussian white noise process, it is

18 $$S_{{\xi_{i} \xi_{j} }} \left( \omega \right) = \mathop {\lim }\limits_{{\hat{T} \to + \infty }} \left\{ {E\left[ {\tilde{\xi }_{i} \left( \omega \right)\tilde{\xi }_{j} \left( \omega \right)} \right]/\hat{T}} \right\} = \mathop {\lim }\limits_{{\hat{T} \to + \infty }} \left\{ {\int\limits_{{ - \frac{{\hat{T}}}{2}}}^{{\frac{{\hat{T}}}{2}}} {\,\,\int\limits_{{ - \frac{{\hat{T}}}{2}}}^{{\frac{{\hat{T}}}{2}}} {E\left[ {\tilde{\xi }_{i} \left( t \right)\tilde{\xi }_{j} \left( {t^{\prime}} \right)} \right]} } \,e^{{ - i\omega (t - t^{\prime})}} dt\,dt^{\prime}} \right\}/\hat{T} = \delta_{ij}$$

Hence the solution components $\tilde{u}\left( \omega \right)$ are given by

19 $$\tilde{u}_{i} \left( \omega \right) = \sum\limits_{j = 1}^{4} {g_{ij} \left( \omega \right)} \,\tilde{\xi }_{j} \left( \omega \right)\,\,,\,\,i = 1,2,3,\,4$$

From (13) we have,

20 $$S_{{u_{i} }} \left( \omega \right) = \sum\limits_{j = 1}^{4} {\beta_{j} \,\left| {g_{ij} \left( \omega \right)} \right|^{2} \,\,,\,\,\,i = 1,2,3} ,\,4$$

Hence the intensities of fluctuations in the variables $u_{i} \,,\,\,\,i = 1,2,3,\,4$ are given by

$$\sigma_{{u_{i} }}^{2} = \left\{ {\sum\limits_{j = 1}^{4} {\int\limits_{ - \infty }^{\infty } {\beta_{j} \left| {g_{ij} (\omega )} \right|^{2} d\omega ;\,\,\,i = 1,2,3} } } \right\}/2\pi ,i = 1,2,3,\,4\,$$

In which, $$g_{mn} (\omega ) = [X_{mn} + iY_{mn} ]/\left| {M\left( \omega \right)} \right|\,;\,\,m,\,n = 1,\,2,\,3,\,4$$

$$X_{11} = \frac{{N_{2} N_{3} N_{4} T^{*} U^{*} }}{{P^{*} S^{{*^{2} }} }} + \frac{{m_{2} N_{3} N_{4} T^{*} U^{{*^{2} }} }}{{P^{*} S^{{*^{2} }} }} - \omega^{2} N_{2} P^{*} - \frac{{\omega^{2} N_{4} U^{*} }}{{P^{{*^{2} }} }} - \frac{{\omega^{2} N_{3} T^{*} }}{{S^{{*^{2} }} }};$$

$$Y_{11} = \frac{{\omega m_{2} N_{4} U^{{*^{2} }} }}{{P^{*} }}\frac{{\omega N_{2} N_{3} T^{*} P^{*} }}{{S^{{*^{2} }} }} - \omega^{3} + \frac{{\omega N_{2} N_{4} U^{*} }}{{P^{*} }} + \frac{{\omega N_{3} N_{4} U^{*} T^{*} }}{{P^{{*^{2} }} S^{{*^{2} }} }};$$

$$X_{12} = 0;Y_{12} = 0;X_{14} = 0;Y_{14} = 0;X_{21} = 0;Y_{21} = 0;X_{23} = 0;Y_{23} = 0;$$

$$X_{13} = - m_{1} S^{*} \left( {\frac{{N_{2} N_{4} U^{*} }}{{P^{*} }} - \omega^{2} + \frac{{m_{2} N_{4} U^{{*^{2} }} }}{{P^{*} }}} \right);$$

$$Y_{13} = - \omega m_{1} S^{*} \left( {N_{2} P^{*} + \frac{{N_{4} U^{*} }}{{P^{{*^{2} }} }}} \right);$$

$$X_{22} = \frac{{m_{1} N_{3} N_{4} T^{{*^{2} }} U^{*} }}{{S^{*} P^{{*^{2} }} }} + \frac{{N_{1} N_{3} N_{4} T^{*} U^{*} }}{{S^{*} P^{{*^{2} }} }}- \frac{{\omega^{2} N_{3} T^{*} }}{{S^{{*^{2} }} }} - \frac{{\omega^{2} N_{4} U^{*} }}{{P^{{*^{2} }} }} - \omega^{2} N_{1} S^{*} ;$$

$$Y_{22} = \frac{{N_{4} N_{3} T^{*} U^{*} \omega }}{{S^{{*^{2} }} P^{{*^{2} }} }} + \frac{{\omega N_{4} N_{1} U^{*} S^{*} }}{{P^{{*^{2} }} }} + \frac{{T^{{*^{2} }} N_{3} \omega m_{1} }}{{S^{*} }} + \frac{{N_{3} N_{1} T^{*} \omega }}{{S^{*} }} - \omega^{3} ;$$

$$X_{24} = - P^{*} m_{2} \left( {\frac{{m_{1} T^{{*^{2} }} N_{3} }}{{S^{*} }} + \frac{{N_{3} N_{1} T^{*} }}{{S^{*} }} - \omega^{2} } \right);Y_{24} = - P^{*} m_{2} \left( {\omega S^{*} N_{1} + \frac{{T^{*} N_{3} \omega }}{{S^{{*^{2} }} }}} \right);$$

$$X_{31} = \frac{{T^{{*^{2} }} N_{3} }}{{S^{{*^{2} }} }}\left( {\frac{{U^{*} N_{4} N_{2} }}{{P^{*} }} + \frac{{U^{{*^{2} }} N_{4} m_{2} }}{{P^{*} }} - \omega^{2} } \right);$$

$$Y_{31} = \frac{{T^{{*^{2} }} N_{3} \omega }}{{S^{{*^{2} }} }}\left( {P^{*} N_{2} + \frac{{U^{*} N_{4} }}{{P^{{*^{2} }} }}} \right);$$

$$X_{32} = Y_{32} = 0;X_{34} = 0 = Y_{34} ;$$

$$X_{33} = \frac{{N_{4} N_{2} N_{1} U^{*} S^{*} a_{44} a_{22} }}{{P^{*} }} + \frac{{U^{{*^{2} }} N_{4} m_{2} }}{{P^{*} }}\, - \frac{{U^{*} N_{4} \omega^{2} }}{{P^{{*^{2} }} }} - P^{*} N_{2} \omega^{2} - S^{*} N_{1} \omega^{2} ;$$

$$Y_{33} = \frac{{S^{*} U^{*} N_{4} N_{1} \omega }}{{P^{{*^{2} }} }} + \frac{{U^{*} N_{4} N_{2} \omega }}{{P^{*} }} + S^{*} P^{*} N_{2} N_{1} \omega - \omega^{3} ;X_{41} = 0;Y_{41} = 0;X_{43} = 0;Y_{43} = 0;$$

$$X_{42} = \frac{{U^{{*^{2} }} N_{4} }}{{P^{*2} }}\left( {\frac{{T^{*} N_{3} N_{1} }}{{S^{*} }} + \frac{{T^{{*^{2} }} N_{3} m_{1} }}{{S^{*} }} - \omega^{2} } \right);$$

$$Y_{42} = \frac{{U^{{*^{2} }} N_{4} \omega }}{{P^{*2} }}\left( {S^{*} N_{1} + \frac{{T^{*} N_{3} }}{{S^{{*^{2} }} }}} \right);$$

$$X_{44} = \frac{{P^{*} T^{{*^{2} }} N_{3} N_{2} m_{1} }}{{S^{*} }} + \frac{{P^{*} T^{*} N_{3} N_{2} N_{1} }}{{S^{*} }} - \frac{{T^{*} N_{3} \omega^{2} }}{{S^{{*^{2} }} }} - P^{*} N_{2} \omega^{2} - S^{*} N_{1} \omega^{2} ;$$

$$Y_{44} = S^{*} P^{*} N_{2} N_{1} \omega + \frac{{T^{{*^{2} }} N_{3} N_{1} \omega }}{{S^{*} }} - \omega^{3} + \frac{{T^{*} N_{3} N_{1} \omega }}{{S^{*} }} + \frac{{P^{*} T^{*} N_{3} N_{2} \omega }}{{S^{{*^{2} }} }};$$

$$\left| {M(\omega )} \right| = R(\omega ) + iI(\omega )$$. Here, $$R(\omega ) = S^{*} X_{31} m_{1} - Y_{11} \omega + S^{*} X_{11} N_{1} ;$$ $$I(\omega ) = S^{*} Y_{31} m_{1} - X_{11} \omega + S^{*} Y_{11} N_{1}$$.

The effect of random environmental fluctuations on specific species can easily be analyzed with either $\beta_{1} = 0$ or $\beta_{2} = 0$ or $\beta_{3} = 0$ or $\beta_{4} = 0$. The analytical evaluation of mean square fluctuations is difficult. But the mean square fluctuations can easily be calculated and visualized for different set of parameters.

**Figure 2:**
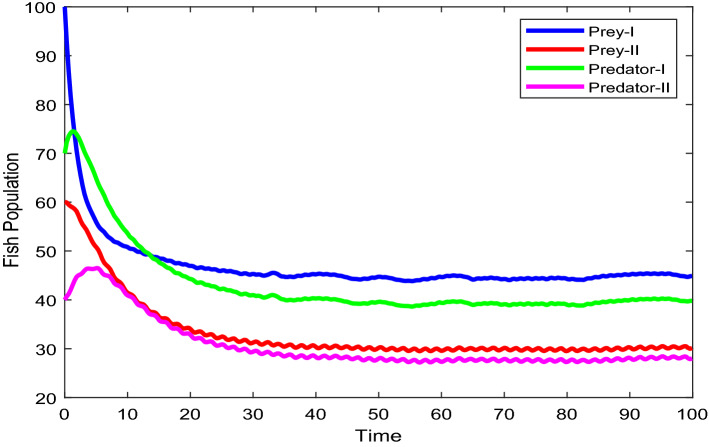
Time series evaluation of fish population for the values of noise intensities of 1.5,1,1.5 and 1 for all 4 category fish population respectively.

**Figure 3:**
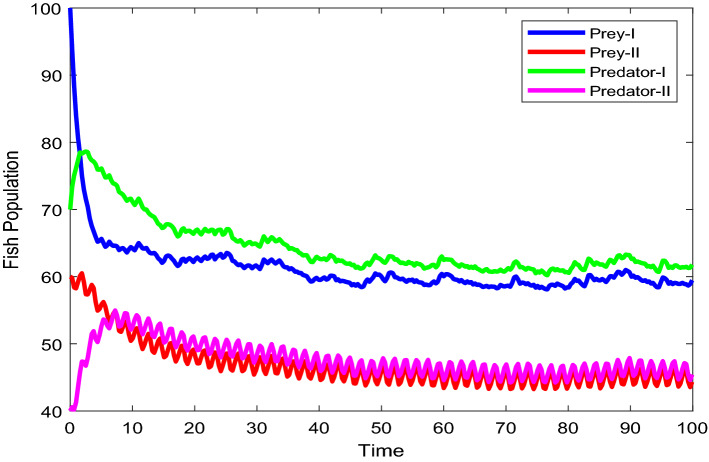
Time series evaluation of populations for the values of noise intensities of 6, 5, 6 and 5 for all 4 category fish population respectively.

**Figure 4:**
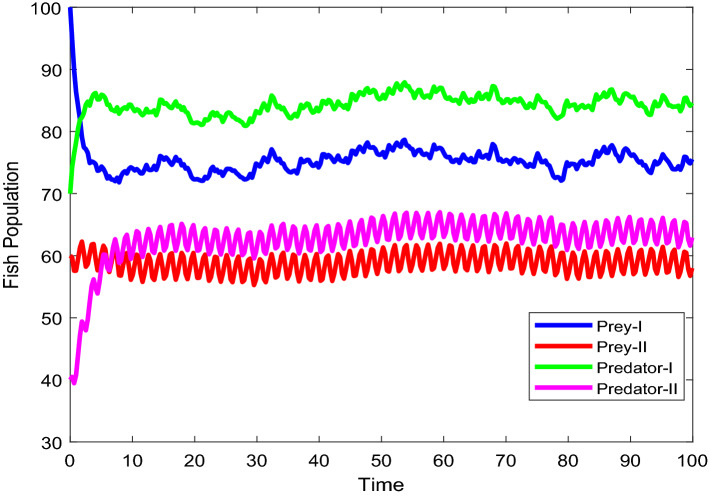
Time series evaluation of populations for the values of noise intensities of 10, 8, 10 and 8 for all 4 category fish population respectively.

**Figure 5:**
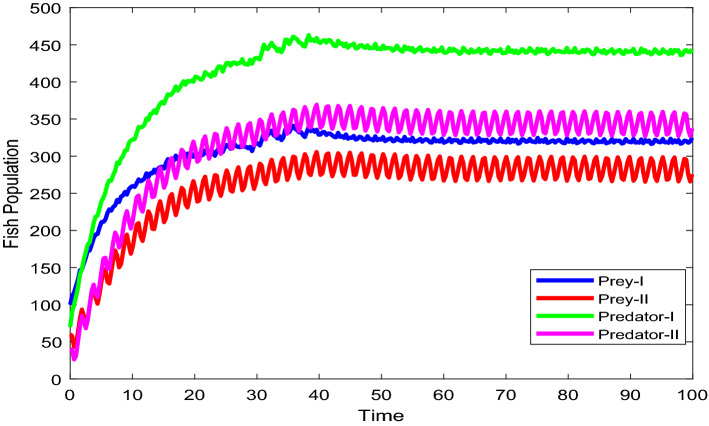
Time series evaluation of populations for the values of noise intensities of 80, 70, 80 and 70 for all 4 category fish population respectively.

**Figure 6:**
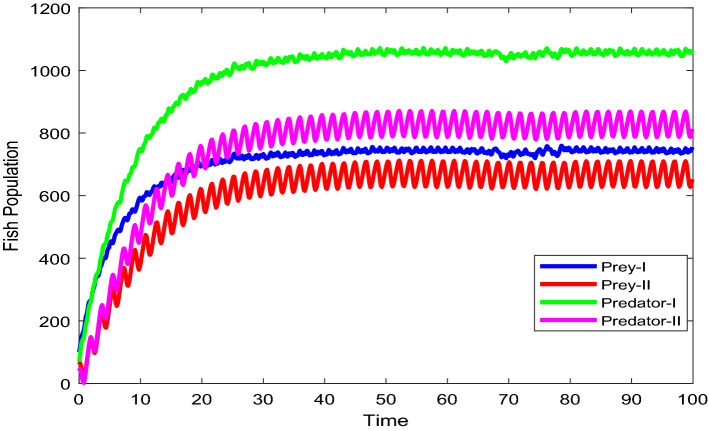
Time series evaluation of populations for the values of noise intensities of 200, 150, 200 and 150 for all 4 category fish population respectively.

Figures 2, 3, 4, 5, 6 are the stochastic graphs for the proposed model with additive noise on fish population named as Prey-I, Prey-II, Predator-I, Predator-II in two patchy environment. Figure 2 represents the time series evaluation of fish population for the values of noise intensities of 1.5, 1, 1.5 and 1 for all 4 category fish population respectively along with the attributes of Table 1. Figure 2 clearly shows the little (very less) oscillatory behavior exhibited by all fish population in two patches, which says model system undergone less influence at these noise intensities (1.5, 1, 1.5, 1). System influenced by additive noise is notable in this Fig. 2. Figure 3 represents the time series evaluation of populations for the values of noise intensities of 6, 5, 6 and 5 for all 4 category fish population respectively along with the attributes of Table 1. Figure 3 clearly shows good oscillatory behavior exhibited by all fish population in two patches, which says model system undergone great influence at these noise intensities (6, 5, 6 and 5). System influenced by additive noise is notable in this Fig. 3. Figure 4 represents the time series evaluation of populations for the values of noise intensities of 10, 8, 10 and 8 for all 4 category fish population respectively along with the attributes of Table 1. Figure 4 clearly shows more oscillatory behavior exhibited by all fish population in two patches, which says model system undergone great influence at these noise intensities (10, 8, 10 and 8). System influenced by additive noise is remarkable in this Fig. 4.

Figure 5 represents the time series evaluation of populations for the values of noise intensities of 80, 70, 80 and 70 for all 4 category fish population respectively along with the attributes of Table 1. Figure 5 clearly shows highly oscillatory behavior exhibited by all fish population in two patches, which says model system undergone high influence at these noise intensities (80, 70, 80 and 70). System influenced by additive noise is highly remarkable in this Fig. 5. Figure 6 represents the time series evaluation of populations for the values of noise intensities of 200, 150, 200 and 150 for all 4 category fish population respectively along with the attributes of Table 1. Figure 6 clearly shows more oscillatory behavior exhibited by all fish population in two patches, which says model system undergone great influence at these noise intensities (200, 150, 200 and 150). System influenced by additive noise is remarkable in this Fig. 6.

### Delay analysis without noise

We looked at a system with two populations in separate patches. The stability of the interior steady state is investigated, and the system is shown to be stable. The stability results have been shown both analytically and quantitatively. We can also use a delayed model system to account for the prey population's gestational delay. It’s only normal that the predator's prey eating takes some time to contribute to the predator's biomass. To investigate such events, we use the delay differential Eqs. (21)–(24).

21 $$x^{\prime}(t) = rx - \sigma_{1} x - q_{1} E_{1} x - m_{1} zx(t - \tau ) - (rx^{2} /K) + \sigma_{2} y$$

22 $$y^{\prime}(t) = \sigma_{1} x + sy - \sigma_{2} y - q_{2} E_{2} y - m_{2} wy(t - \tau ) - (sy^{2} /L)$$

23 $$z^{\prime}(t) = \alpha_{1} z - \left( {\alpha_{1} z^{2} /\gamma_{1} x} \right)$$

24 $$w^{\prime}(t) = \alpha_{2} w - \left( {\alpha_{2} w^{2} /\gamma_{2} y} \right)$$

Das and Gazi^31,32^ have studied delay differential equation models extensively in the study of numerous ecological systems. All of such systems are two-dimensional and three-dimensional. The analytical examination of the system will be difficult to tractable since the current model is a four-dimensional system, and the expression for the delay parameter values will be convoluted for which the system is stable. As a result, we only solve the system numerically.

### Numerical simulations

**Figure 7:**
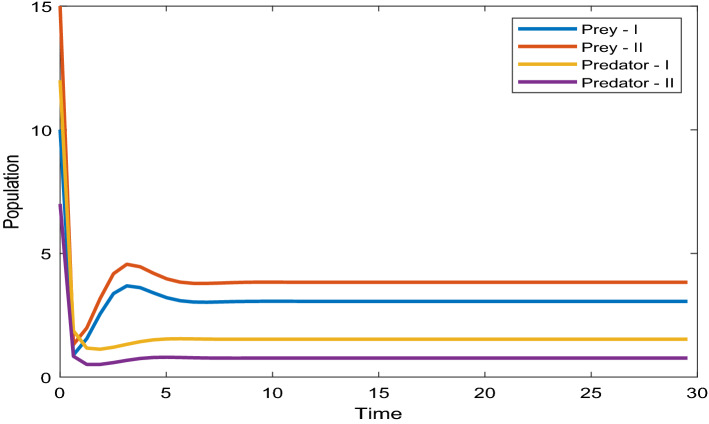
Time series evaluation of population (Prey—I, Prey—II, Predator—I, Predator—II) with initial values^7,10,12,15^.

The following set of parameters is used for the Figs. 1, 2, 3, 4, 5, 6, 7 to analyze the simulation: $r = 3$, $s = 3.2$, $\,\sigma_{1} = 1.8$, $\sigma_{2} = 1.6$, $m_{2} = 1.8$, $m_{1} = 1.2$, $K = 10$, $L\, = 15$, $\gamma_{1} = 0.5\,,$ $\gamma_{2} = 0.2,\,$ $q_{1} = 0.5,$ $q_{2} = 0.7,$ $E_{1} = 0.9$, $E_{2} = 1.2$, $\alpha_{1} = 0.5$, $\alpha_{2} = 0.8$.

Figure 7 shows that the time series evaluation dynamics of populations for different initial values^7,10,12,15^. It is clear from the figures that the system exhibits steadiness with in a very short span of time and the system comes to steadiness after certain period of time. They are varying with time for different initial values.

**Figure 8:**
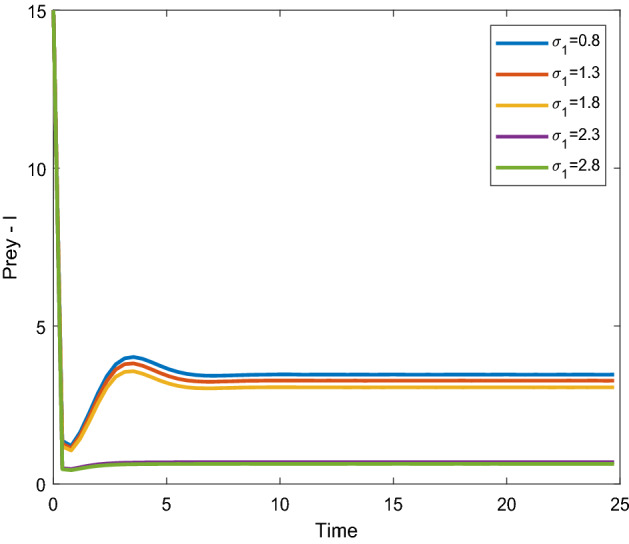
Time series evaluation of population (prey–I) for various values of ($\sigma_{1}$) $\sigma_{1} = \,\,0.8\,\,;\,\,\sigma_{1} = \,\,1.3\,\,;\,\,\sigma_{1} \, = \,\,1.8\,\,;\,\,\sigma_{1} \, = \,\,2.3\,\,;\,\,\sigma_{1} \, = \,2.8\,\,$.

**Figure 9:**
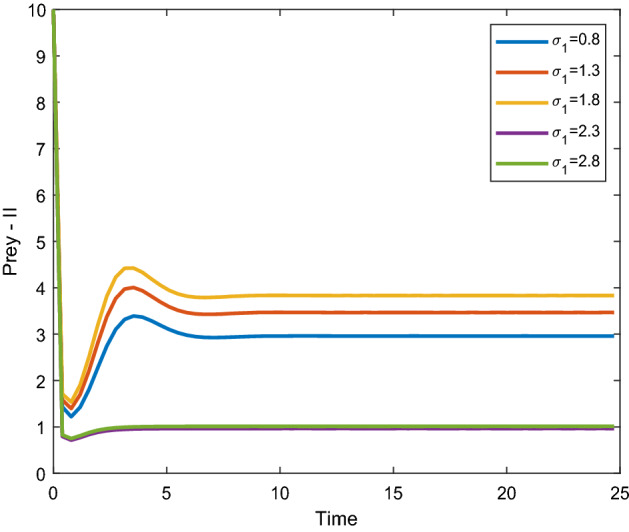
Time series evaluation of population (prey-II) for various values of ($\sigma_{1}$) $\sigma_{1} = \,\,0.8\,\,;\,\,\sigma_{1} = \,\,1.3\,\,;\,\,\sigma_{1} \, = \,\,1.8\,\,;\,\,\sigma_{1} \, = \,\,2.3\,\,;\,\,\sigma_{1} \, = \,2.8\,\,$.

**Figure 10:**
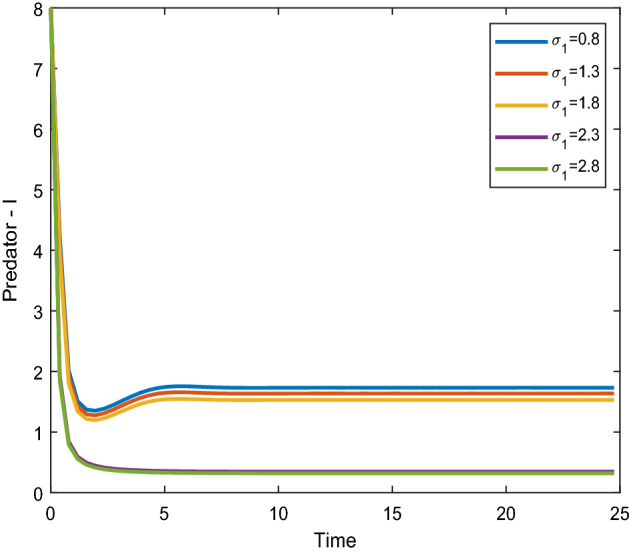
Time series evaluation of population (predator–I) for various values of ($$\sigma_{1}$$) $$\sigma_{1} = \,\,0.8\,\,;\,$$$$\sigma_{1} = \,\,1.3\,\,;$$ $$\sigma_{1} \, = \,\,1.8\,\,;$$ $$\sigma_{1} \, = \,\,2.3\,\,;\,\,\sigma_{1} \, = \,2.8\,\,$$.

**Figure 11:**
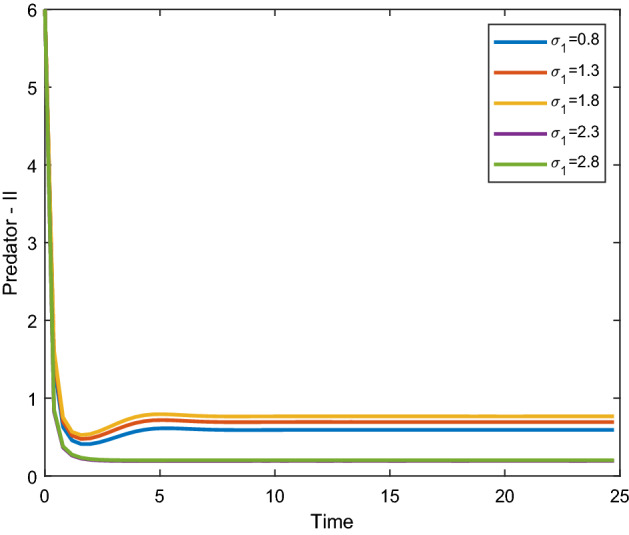
Time series evaluation of population (predator-II) for various values of ($$\sigma_{1}$$) $$\sigma_{1} = \,\,0.8\,\,;\,$$$$\sigma_{1} = \,\,1.3\,\,;$$ $$\sigma_{1} \, = \,\,1.8\,\,;$$ $$\sigma_{1} \, = \,\,2.3\,\,;\,\,\sigma_{1} \, = \,2.8\,\,$$.

Figures 8, 9 shows that the time series evaluation dynamics for both prey populations. And Figs. 10, 11 shows the time series evaluation dynamics of both predator populations for different values of migration rate from parch-1 to patch-2($\sigma_{1}$). It is clear from the figures that the system exhibits steadiness with in a very short span of time and the system comes to steadiness after certain period of time. They may vary with for different initial values.

**Figure 12:**
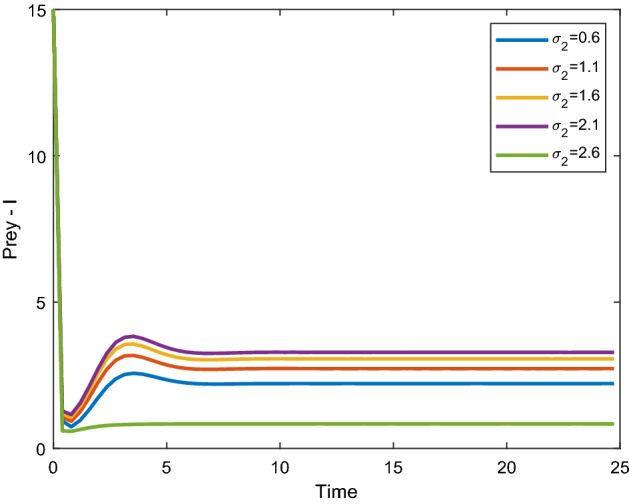
Time series evaluation of population (prey–I) for various values of ($\sigma_{2}$). $\sigma_{2} = \,\,0.6\,\,;\,\,\sigma_{2} = \,\,1.1\,\,;\,\,\sigma_{2} \, = \,\,1.6\,\,;\,\,\sigma_{2} \, = \,\,2.1\,\,;\,\,\sigma_{2} \, = \,2.6\,\,$.

**Figure 13:**
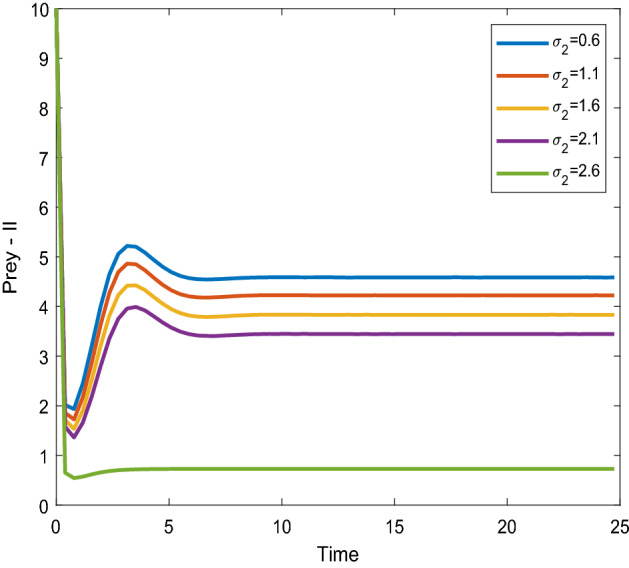
Time series evaluation of population (prey-II) for various values of ($\sigma_{2}$). $\sigma_{2} = \,\,0.6\,\,;\,\,\sigma_{2} = \,\,1.1\,\,;\,\,\sigma_{2} \, = \,\,1.6\,\,;\,\,\sigma_{2} \, = \,\,2.1\,\,;\,\,\sigma_{2} \, = \,2.6\,\,$.

**Figure 14:**
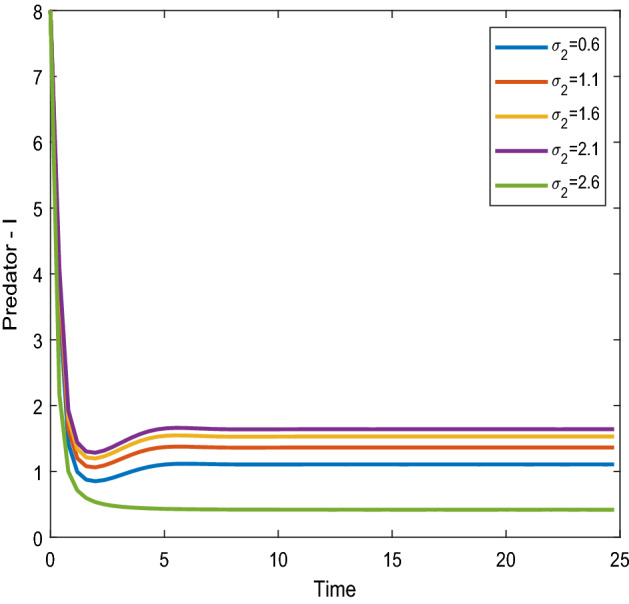
Time series evaluation of population (predator–I) for various values of $\sigma_{2} = \,\,0.6\,\,;\,\,\sigma_{2} = \,\,1.1\,\,;\,\,\sigma_{2} \, = \,\,1.6\,\,;\,\,\sigma_{2} \, = \,\,2.1\,\,;\,\,\sigma_{2} \, = \,2.6\,\,$.

**Figure 15:**
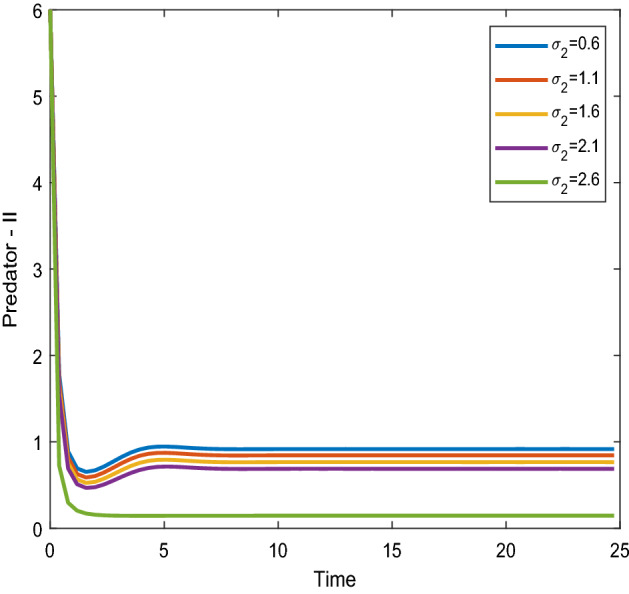
Time series evaluation of population (predator-II) for various values of $\sigma_{2} = \,\,0.6\,\,;\,\,\sigma_{2} = \,\,1.1\,\,;\,\,\sigma_{2} \, = \,\,1.6\,\,;\,\,\sigma_{2} \, = \,\,2.1\,\,;\,\,\sigma_{2} \, = \,2.6\,\,$.

Figures 12, 13 shows that the time series evaluation dynamics for both prey populations. And Figs. 14, 15 shows the time series evaluation dynamics of both predator populations for different values of migration rate from parch-2 to patch-1($\sigma_{2}$). These figures clearly show that the system demonstrates steadiness within a very short length of time and that it attains steadiness after a specific amount of time. They change throughout time depending on the original initial numeric.

**Figure 16:**
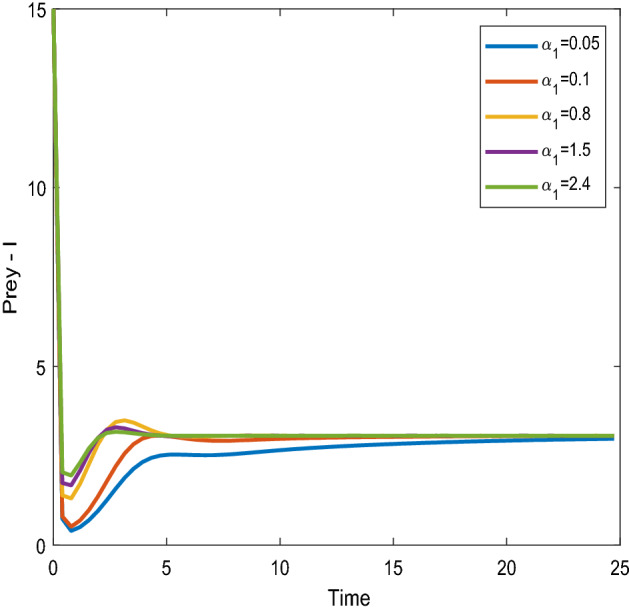
Time series evaluation of population (prey–1) for various values of ($\alpha_{1}$) $\alpha_{1} = \,\,0.05\,\,;\,\alpha_{1} = \,\,0.1\,\,;\,\alpha_{1} = \,\,0.8\,\,;\,\alpha_{1} = \,\,1.5\,;\,\alpha_{1} = \,\,2.4\,$.

**Figure 17:**
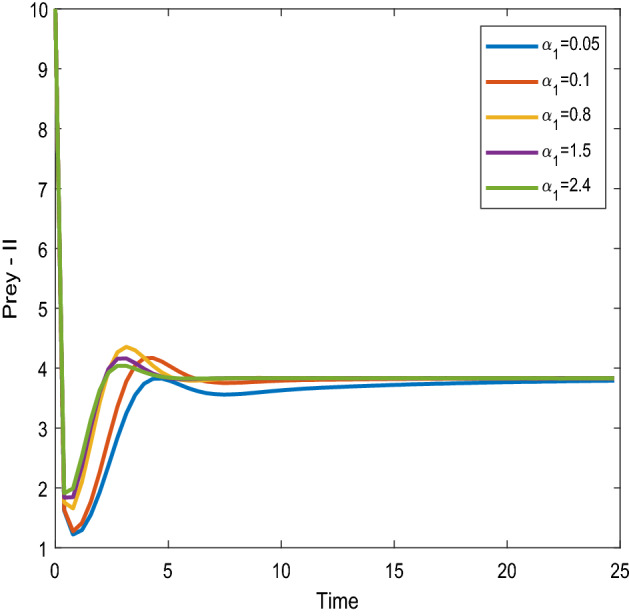
Time series evaluation of population (prey-II) for various values of ($\alpha_{1}$) $\alpha_{1} = \,\,0.05\,\,;\,\alpha_{1} = \,\,0.1\,\,;\,\alpha_{1} = \,\,0.8\,\,;\,\alpha_{1} = \,\,1.5\,;\,\alpha_{1} = \,\,2.4\,$.

**Figure 18:**
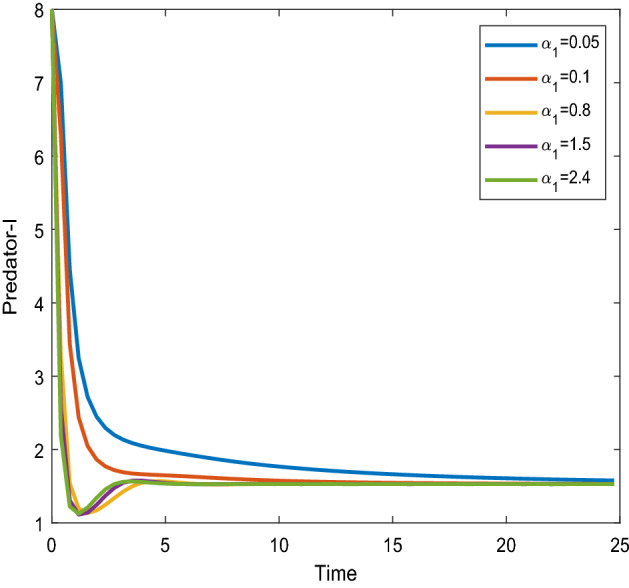
Time series evaluation of population (predator-I) for various values of ($\alpha_{1}$) $\alpha_{1} = \,\,0.05\,\,;\,\alpha_{1} = \,\,0.1\,\,;\,\alpha_{1} = \,\,0.8\,\,;\,\alpha_{1} = \,\,1.5\,;\,\alpha_{1} = \,\,2.4\,$.

**Figure 19:**
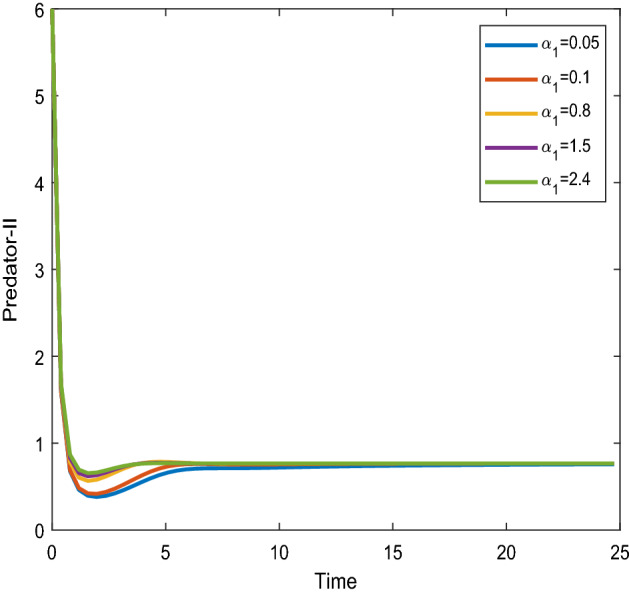
Time series evaluation of population) (predator-II) for various values of ($\alpha_{1}$) $\alpha_{1} = \,\,0.05\,\,;\,\alpha_{1} = \,\,0.1\,\,;\,\alpha_{1} = \,\,0.8\,\,;\,\alpha_{1} = \,\,1.5\,;\,\alpha_{1} = \,\,2.4\,$.

Figures 16, 17 shows that the time series evaluation dynamics for both prey populations. And Figs. 18, 19 shows the time series evaluation dynamics of both predator populations for different values of intrinsic growth rate of predator species in patch-1($\alpha_{1}$). These figures clearly show that the system demonstrates steadiness within a very short length of time and that it attains steadiness after a specific amount of time. Additionally, there is a minor partial decline in the densities of both the prey populations (patch-1).

**Figure 20:**
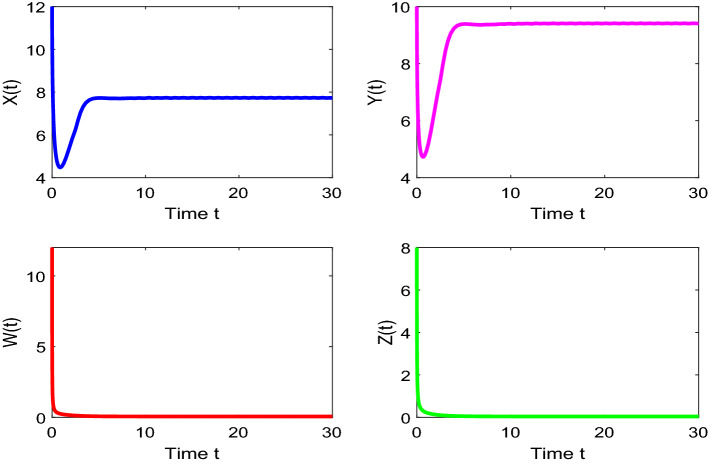
Time series graph for the population prey-I, prey-II, predator-I and predator-II at the delay values $\tau_{1} = 2.5$.

**Figure 21:**
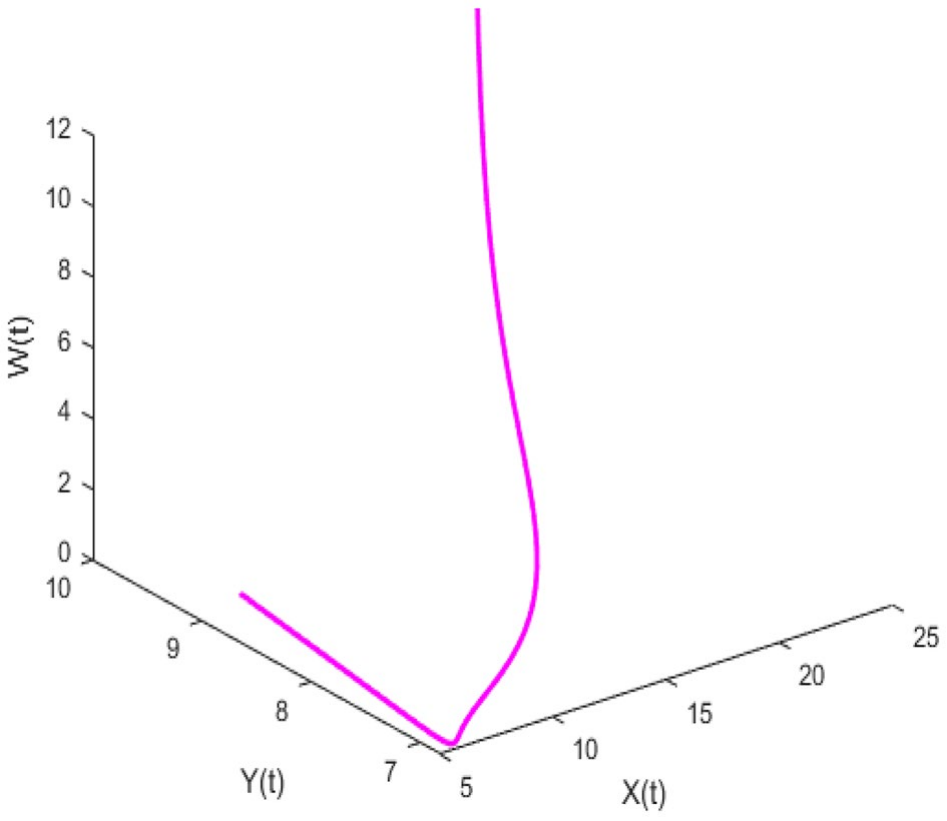
Phase portrait plot for the population (prey-I, prey-II and predator-I) respectively at the delay values $\tau_{1} = 2.5$.

**Figure 22:**
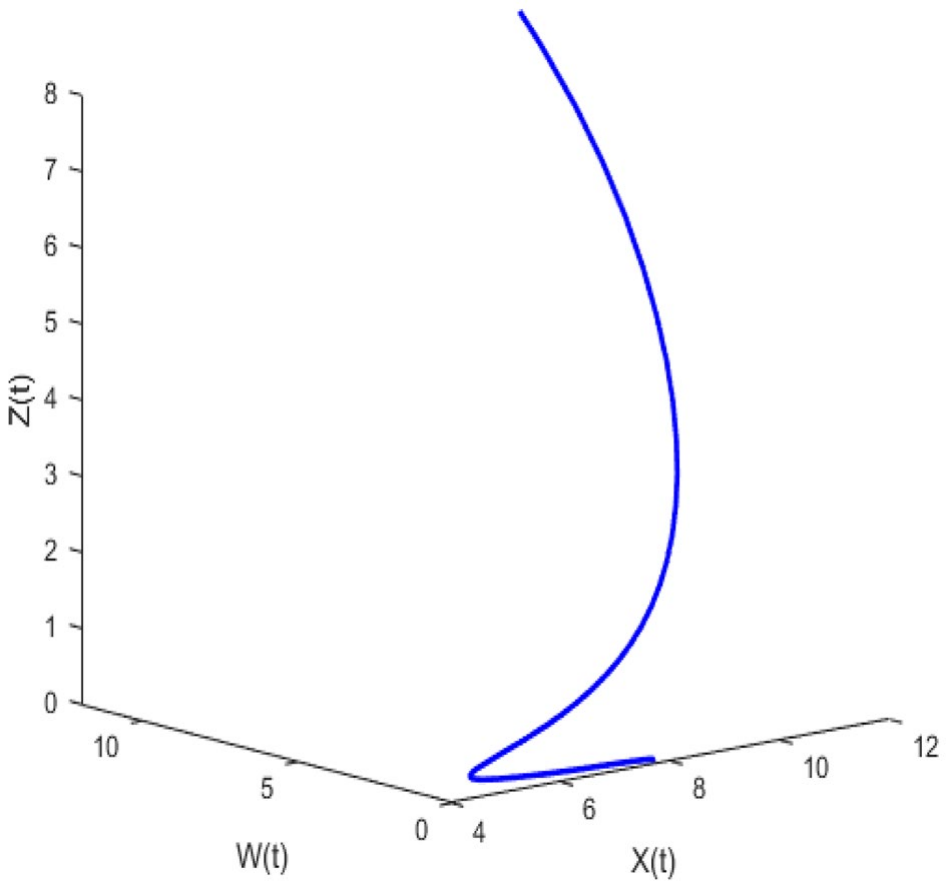
Phase portrait plot for the population (Prey-I, predator-I and predator-II) respectively at the delay values $\tau_{1} = 2.5$.

**Figure 23:**
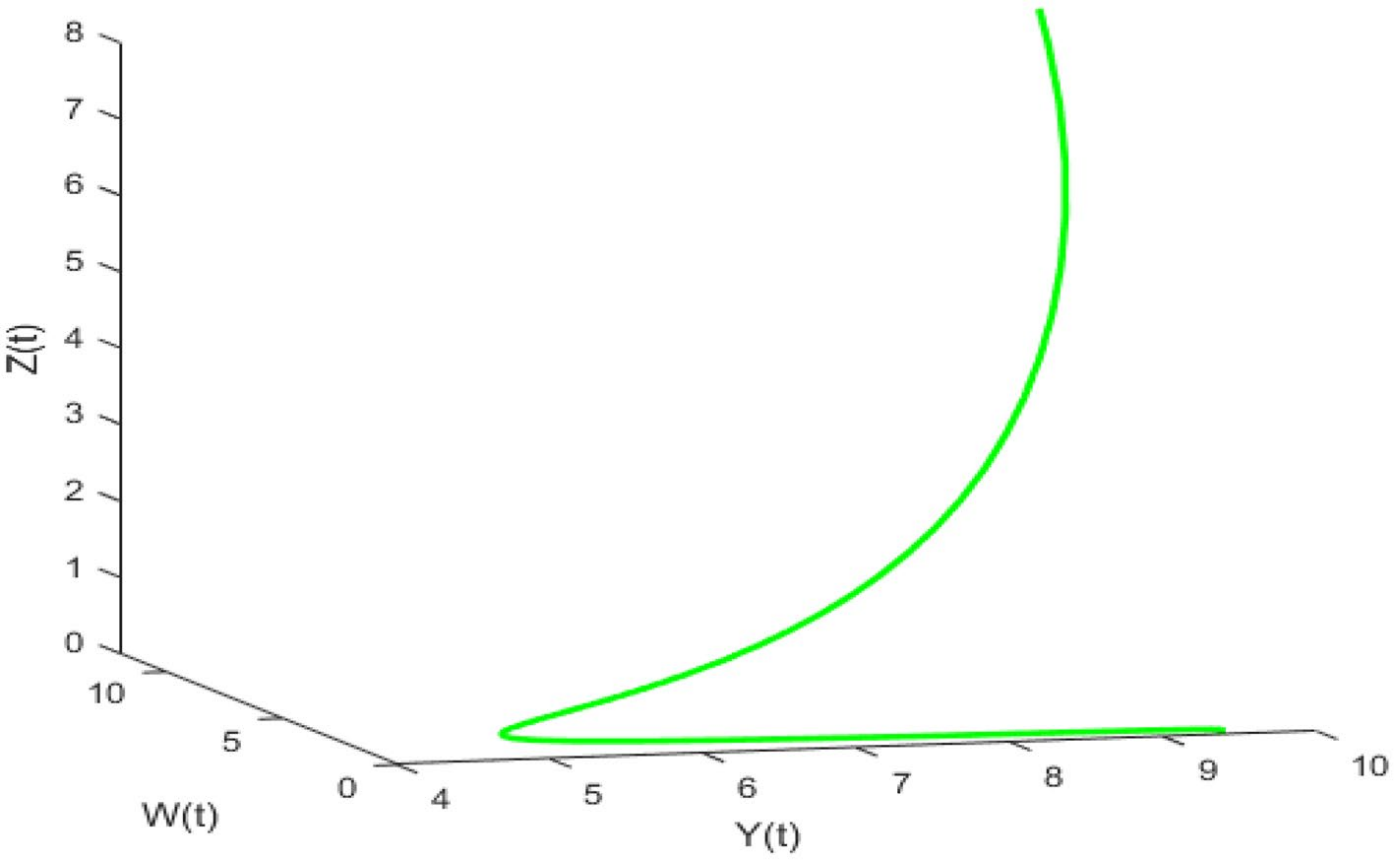
Phase portrait plot for the population prey-II, predator-I and predator-II at the delay values $\tau_{1} = 2.5$.

Figure 20 depicts the population time series evaluation at a delay value $\tau_{1} = 2.5$. This figure clearly shows that, in contrast to the predator populations of the system, the prey populations of the system exhibit stability within a very short length of time and come to steadiness after a specific amount of time. The beginning values will determine whether they alter over time. Figures 21, 22, and 23 displays phase portrait diagrams for the populations of the proposed system (X(t), Y(t), W(t); X(t), W(t), Z(t); and Y(t), W(t), Z(t)). They might change throughout time depending on the initial settings of numeric.

Figure 25 depicts the population time series evaluation at a delay value $\tau_{1} = 12.5$, which clearly shows that, all populations of the system, exhibit oscillatory behavior and unstable nature.

Figures 26, 27, 28, 29 presents phase portrait projections plotted among X(t), Y(t), Z(t) and W(t) at a delay value $\tau_{1} = 11.5$, which clearly shows that, limit cycle oscillations of the system and converges to a stable equilibrium point.

## Concluding remarks

With prey migration and prey harvesting, the two-patch aquatic habitat for prey and predator is explored in this paper. The growth of the species is considered in a logistical approach. Because the model is four-dimensional, there are 16 mathematical equilibrium points, but the presence of all species and their dynamics is of great ecological significance. As a result, the stability study is limited to the inner steady state. Interior steady state expressions and existence conditions are derived. Routh-Hurwitz criteria are used to determine the system's local stability behaviour in the absence of noise. The Lyapunov theorem is used to investigate the global dynamics of the system in the absence of noise around the interior equilibrium point. The prey population intervals produced show that the system may be kept globally asymptotically stable if both prey populations are within a given range. Additive white noise perturbations following a Gaussian process are used to approximate random environmental disturbances. The higher the population variance, the more unstable the system is, and the lower the population variance, the better the stochastic system's stability behaviour in the respective area. The analytical conclusions may be confirmed using numerical simulations as a future scope of the current analysis. Distinct sets of parameters can display different stability behaviours in local and global aspects, and numerical simulations can easily identify the model's sensitive parameters. The system behaviour with fluctuations in population densities can be simulated and compared for different sets of noise amplitudes. Furthermore, ideal harvesting levels for prey species can be determined in order to promote species expansion and earnings.

Delay induced system analysed both analytically and graphically, delay dynamics are identified, analysed and presented well with the help of numerical simulations.

Figure 20 shows the population time series evaluation at a delay value $\tau_{1} = 2.5$, which clearly shows that, all populations of the system, exhibit stable nature very soon. All Populations- X(t), Y(t), Z(t) and W(t) attains stability very soon and the system is not completely influenced by delay parameter at this value $\tau_{1} = 2.5$. Prey-I, Prey-II shows very little variation at this delay value and Predator-I, Predator-II are not under the influence of delay at this value.

**Figure 24:**
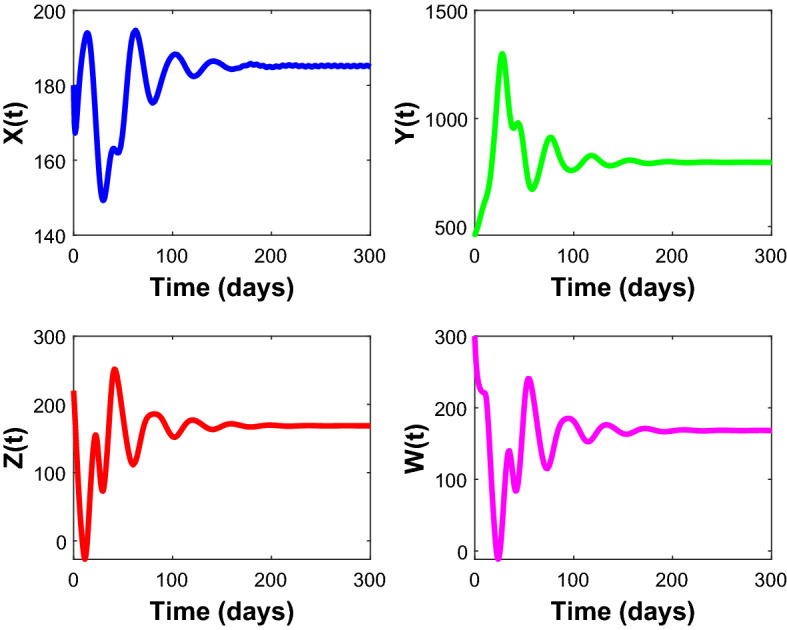
Time series graph for the population X(t)(Prey-I), Y(t)(Prey-II), Z(t)(Predator-I) and W(t)(Predator-II) at the delay value $\tau_{1} = 10.5$.

**Figure 25:**
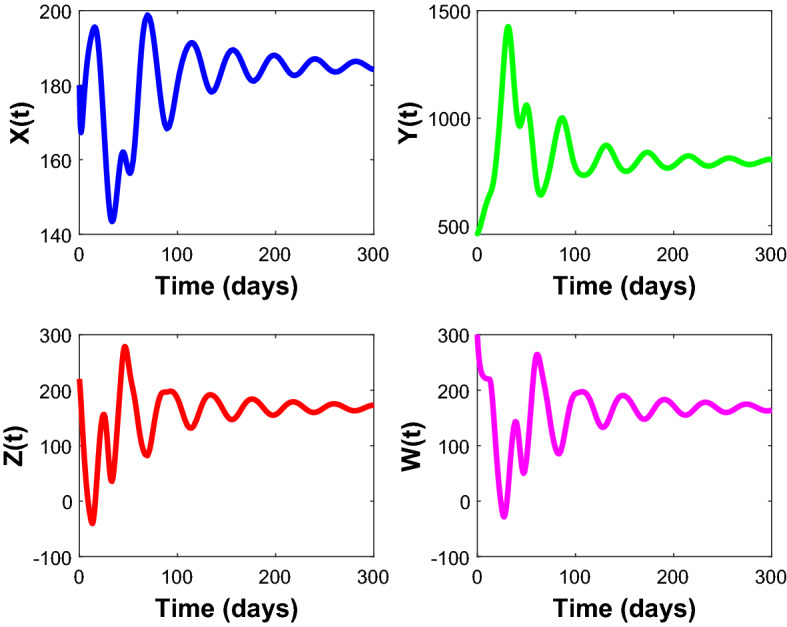
Time series graph for the population X(t)(Prey-I), Y(t)(Prey-II), Z(t)(Predator-I) and W(t)(Predator-II) at the delay value $\tau_{1} = 12.5$.

**Figure 26:**
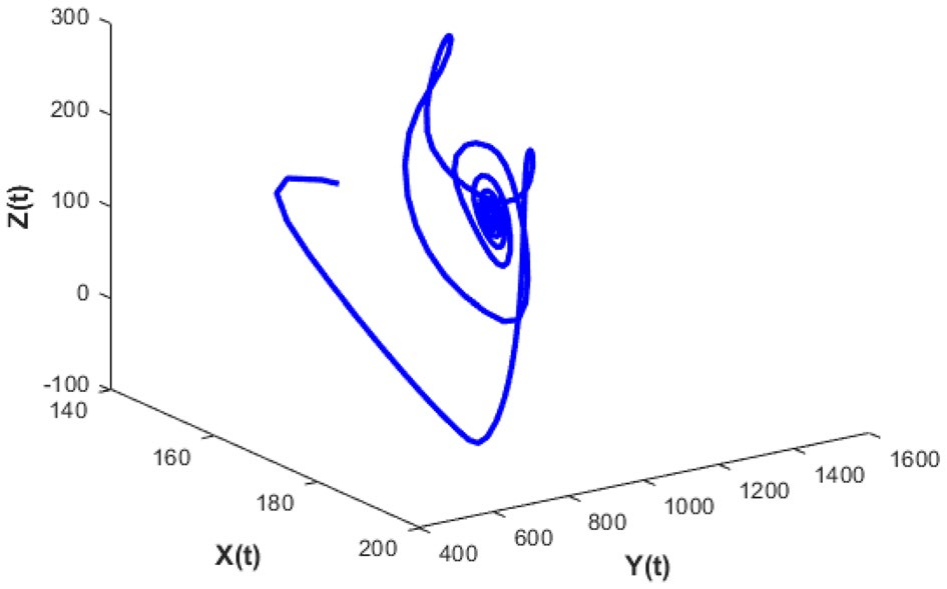
Phase portrait plot for the population (Prey-I, Prey-II and Predator-I) respectively at the delay values $\tau_{1} = 11.5$.

**Figure 27:**
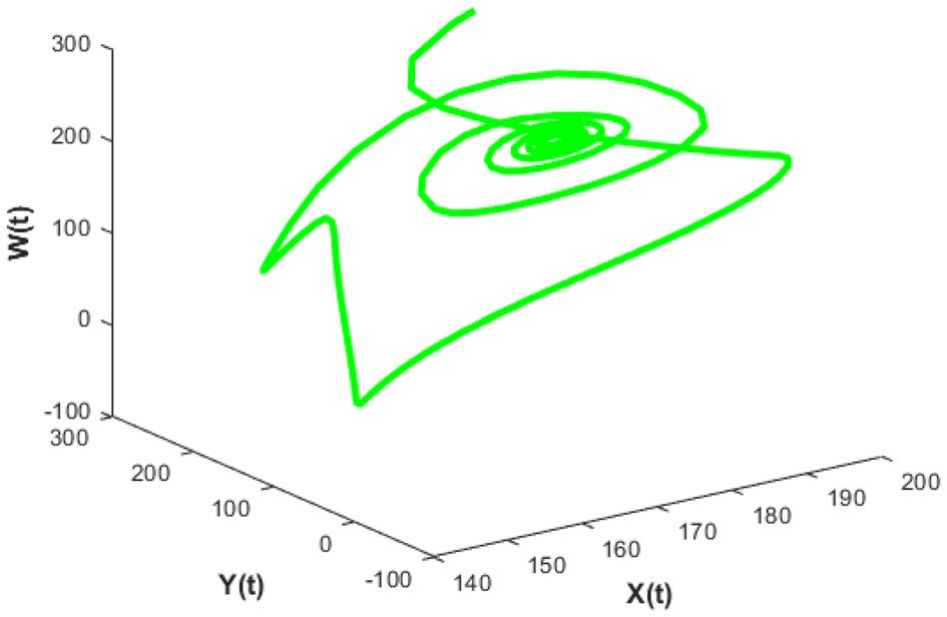
Phase portrait plot for the population (Prey-I, Prey-II and Predator-II) respectively at the delay values $\tau_{1} = 11.5$.

**Figure 28:**
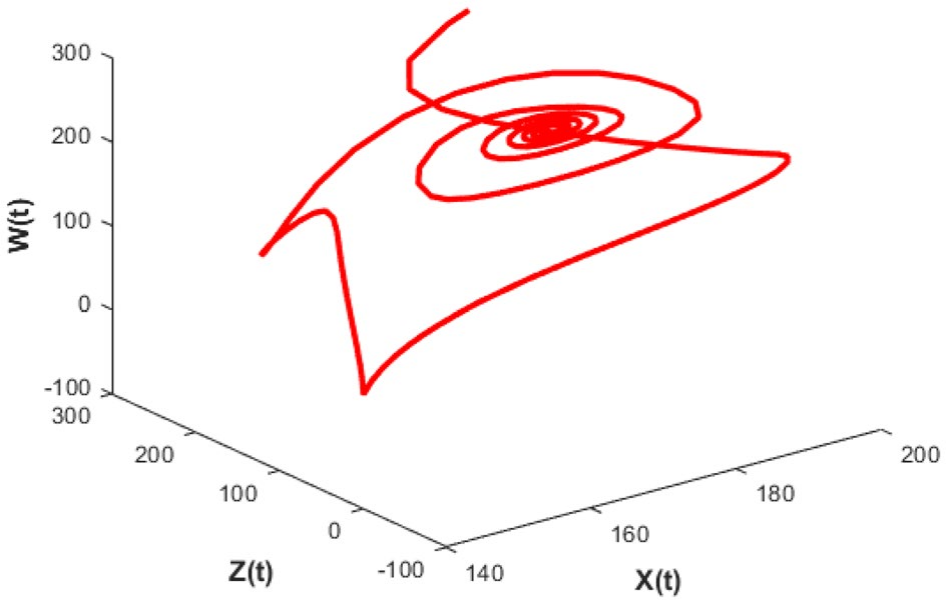
Phase portrait plot for the population (prey-I, predator-I and predator-II) respectively at the delay values $\tau_{1} = 11.5$.

**Figure 29:**
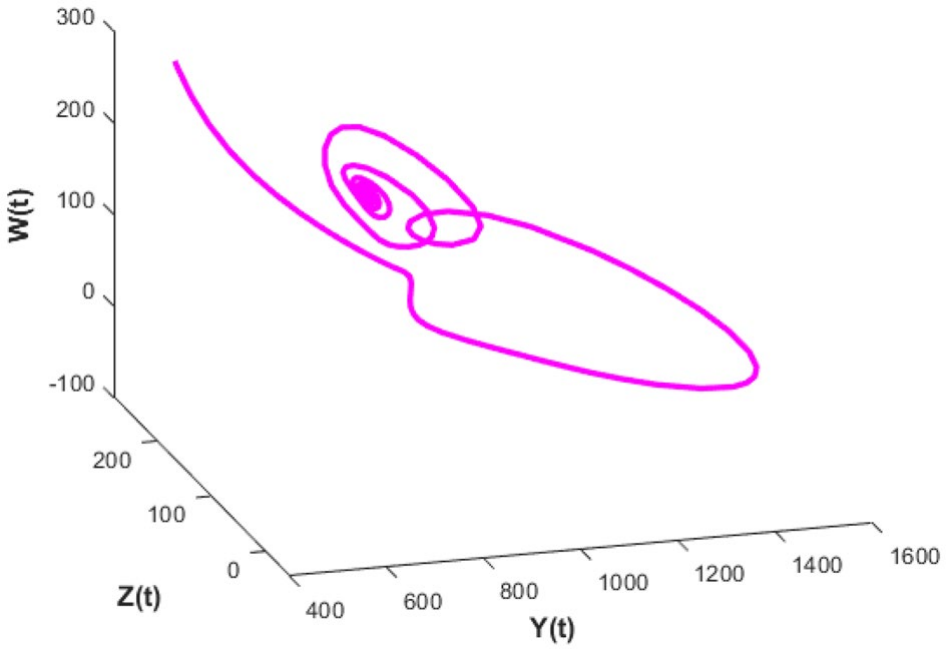
Phase portrait plot for the population (Prey-II, predator-I and predator-II) respectively at the delay values $\tau_{1} = 11.5$.

Figure 24 depicts the population time series evaluation at a delay value $\tau_{1} = 10.5$, which clearly shows that, all populations of the system, exhibit little oscillatory for some period of time and after certain period of time all the populations X(t), Y(t), Z(t) and W(t) attains stability. Which says that system undergone the good effect of delay, which is exhibited by its oscillatory behavior. Figure 25 depicts the population time series evaluation at a delay value $\tau_{1} = 12.5$, which clearly shows that, all populations of the system, exhibit gradual and consistent oscillatory and unstable nature. At this value of delay $\tau_{1} = 12.5$ all the populations X(t), Y(t), Z(t) and W(t) undergone high influence delay and shown their oscillatory behavior. Which says that system undergone the influence of delay highly, which is exhibited by its continued oscillatory behavior. Figures 26, 27, 28, 29 depicts the population time series evaluation at a delay value $\tau_{1} = 11.5$, which clearly shows that, limit cycle oscillations of the system and converges to a stable equilibrium point. Figures 2, 3, 4, 5, 6 depicts the time series plots for the stochastic model (1)–(4) System undergone the influence of Gaussian white noise, exhibits its dynamics in the form of chaotic and oscillatory nature in the graphical solutions. Prey- 1 and Predator-1 are less oscillatory and Prey-2 and Predator-2 Parameter analysis is carried for influential attributes in the model (1)–(4) and the values of all attributes are from Table 1.

In this paper, we studied the delay dynamics and stochastic dynamics in view of fishery resource management along with migration of prey species in both the zones (patches). This work is also connected to the impact of harvesting and migration (allowed for prey in both patches) on the proposed model. Few examples on prey-1 and prey-2 are Gold band fish/small pelagic fish/small tunas/shrimps. Few examples on predator-1 and predator-2 are shark fish/Dolphin fish/Great white shark/stone fishes etc.

## Data Availability

All data generated or analyzed during the study are included in this article.
